# The anterior transmuscular intrapelvic approach for the treatment of acetabular fractures—a new anterior surgical strategy

**DOI:** 10.1186/s12891-023-06775-2

**Published:** 2023-08-09

**Authors:** Sebastian Lippross, Clara Wehrenpfennig, Thilo Wedel, Andreas Seekamp, Daniar Osmonov, Babak Moradi, Stefanie Fitschen-Oestern, Joerg Finn, Tim Klueter, Bodo Kurz, Ibrahim Alkatout

**Affiliations:** 1 Department of Trauma and Orthopaedic Surgery, University Medical Center of Schleswig-Holstein, Campus Kiel, Kiel, Germany; 2 Institute of Anatomy, Christian-Albrecht University, Kiel, Germany; 3 Department of Urology, University Medical Center of Schleswig-Holstein, Campus Kiel, Kiel, Germany; 4 Department of Gynaecology, University Medical Center of Schleswig-Holstein, Campus Kiel, Kiel, Germany

**Keywords:** Anterior intrapelvic approach, Acetabular fracture

## Abstract

The anterior ilioinguinal and the posterior Kocher-Langenbeck approach have long been the standard surgical approaches to the acetabulum. The last decade has witnessed the development of so-called intrapelvic approaches for anterior pathologies because they provide better exposure of the quadrilateral plate. Currently, the modified Stoppa approach and the pararectus approach are frequently used by surgeons for the treatment of acetabular fractures. We investigated an even more direct access to the entire anterior column and the quadrilateral plate via the abdominal wall muscles, between the incisions for the ilioinguinal and the pararectus approach.

After intensive study of anatomic specimens, a cadaver dissection was performed prior to clinical application. The approach was then used in 20 patients who were assessed retrospectively.

Postoperative CT scans showed that, according to the Matta scoring system, the quality of fracture reduction was “anatomical” (≤ 1 mm) in 12 (60%), “imperfect” (2–3 mm) in four (20%), and “poor” (> 3 mm) in four (20%) patients. Numerous minor complications were observed; the majority of these had resolved at the time of discharge.

In conclusion, the anterior transmuscular intrapelvic approach (ATI) is a safe and effective alternative to the ilioinguinal and pararectal approaches, and may be regarded as an evolutionary advancement of traditional procedures.

## Introduction

Since the establishment of the Kocher-Langenbeck and the ilioinguinal approach, surgery has become the standard procedure for the treatment of displaced acetabular fractures [1, 2]. However, we lack consensus as to the choice of procedure for each type of fracture. Traditionally, Letournel´s classification of acetabular fractures consisting of five simple and five complex fracture patterns is used by most surgeons [3]. Given the increasing elderly population and the frequency of acetabular fractures due to osteoporosis, the involvement of the quadrilateral plate deserves attention. Central protrusion of the femoral head with dislocation of the anterior column (as part of both-column, anterior column and posterior hemitransverse, and T-type fractures) has emerged as a common fracture pattern [4].

The ilioinguinal approach includes anatomical dissection of the upper and outer and parts of the ilium, allowing for excellent reduction of the anterior column. The approach has been used successfully for many years and still serves as a reliable procedure when performed by an experienced surgeon. The modified Stoppa approach and the pararectus approach are now frequently used to address anterior pathologies and directly visualize the quadrilateral plate [5–7]. Both approaches permit dissection from the pubic symphysis to the sacroiliac joint, and both may be used to address fractures extending into the ilium.

Recently, Chen et al. reported a novel utilitarian approach [8] with a surgical route straight through the three layers of the abdominal wall, lateral to the pararectus and medial to the ilioinguinal route. This procedure is believed to provide the most direct access to the pelvic viscera, the ilium, and the quadrilateral plate. According to proponents of the approach, cutting muscular tissue rather than the fascia that connect groups of muscles provides better healing and is associated with fewer soft tissue complications.

We performed an anatomical study of the proposed approach compared to standard approaches, combining orthopedic, gynecological, urological and anatomical expertise.

## Materials and methods

### Body donors and patients

The cadavers were fixed in an ethanol-glycerol-lysoformin solution as described previously [9], which provided an optimum consistency and permitted easy handling of soft tissue.

## Surgical procedure

### Access route

**Fig. 1:**
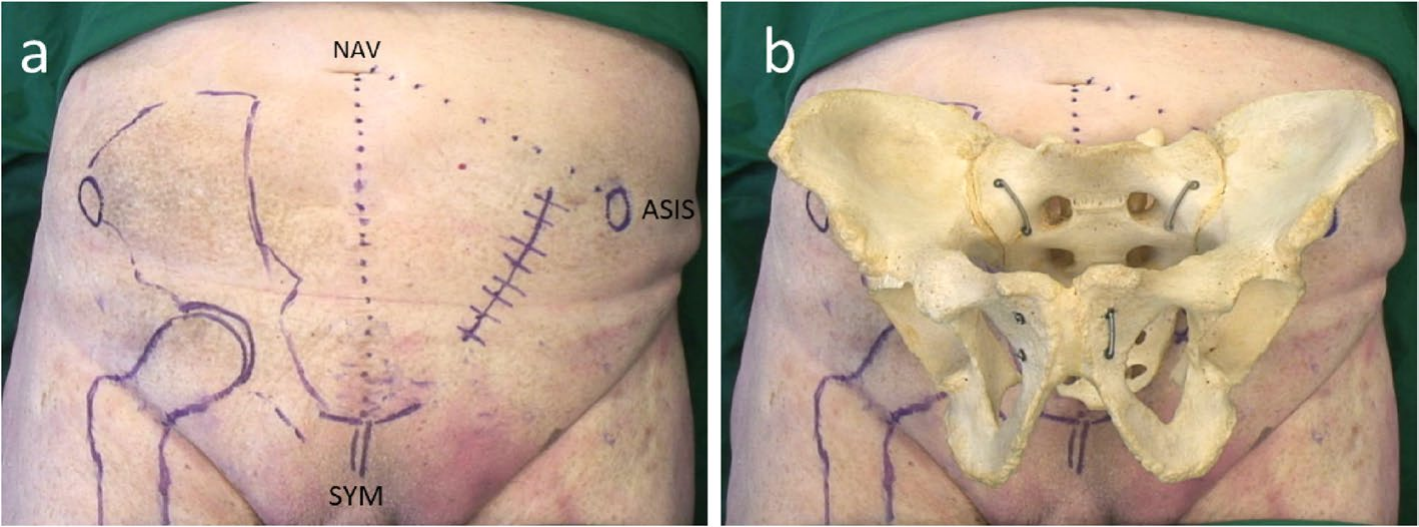
Anatomic landmarks and topography of the abdomen. **a** Two fingerbreadths medial to the ASIS an incision is made slightly curved and towards the pubic symphysis (ASIS anterior superior iliac spine, SY symphysis, NAV naval). **b** illustration of relation to the underlying bony anatomy. The incision lies directly over the acetabulum.Dissection of subcutaneous fat was followed by exposure and splitting of Scarpa’s fascia and the muscle fascia of the external abdominal oblique muscle in line with the skin incision (Fig. 2a). All three lateral abdominal muscles were divided according to their natural course, while the thin fasciae of the internal oblique and transverse abdominal muscles and the transversalis fascia were cut in line with the skin incision

The body donor/patient was positioned supine with a cushion roll beneath the knee to relax the hip flexors on the injured side. The landmarks, i.e. the anterior superior iliac spine (ASIS) and the midline through the navel and pubic symphysis were identified. The incision was started two fingerbreadths medial to the ASIS, and performed in a straight line (about 10 cm) towards the pubic symphysis (Fig. 1).

**Fig. 2:**
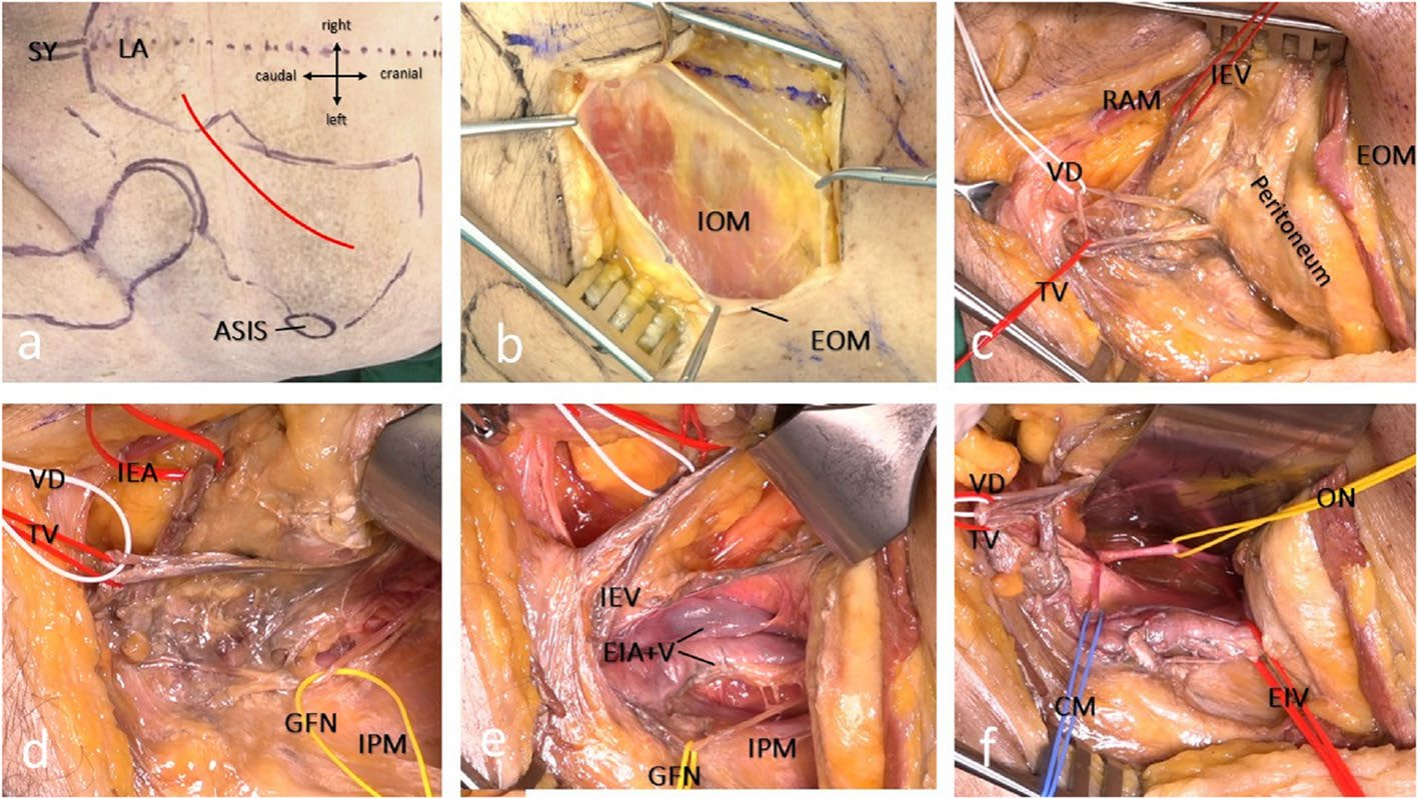
**a** Surgeons view from the left side, Incision (red line) in relation to the anatomic landmarks (ASIS anterior superior iliac spine, SY symphysis). **b** 3 layers of the abdominal wall can be split in line with the fibers of the muscle (IOM internal oblique muscle, EOM external oblique muscle). **c** Just below the internus abdominis fascia lies the bundle of the inferior epigastric vessels (IEV) that guide the surgeon to the external iliac vessels. All structures are embedded by fat and lymphatic tissue. Careful dissection reveals the vas deferens (VD) and accompanying deferential vessels (DV). **d** The genitofemoral nerve (GFN) lies on the iliopsoas muscle (IPM). To expose the iliac wing and the posterior part of the iliopubic line, the IPM can be retracted medially together with the GFN. **e** careful dissection along the Inferior epigastric vessels will lead to exposure of the external iliac artery and vein (EIA + V). Lateral to this lies the medial window, laterally bordered by IPM, allowing access to the acetabular roof and the iliopectineal line. **f** lateral Retraction of the IEA + V opens the median window. Mostly a corona mortis (CM) will be visible. Blunt dissection along the quadrilateral plate will reveal the obturator nerve (ON) and accompanying vessels

The inferior epigastric vessels originating from the external iliac artery and vein were identified (Fig. 2b) in the dorsal aspect of the last fascial layer, before they headed towards the posterior aspect of the rectus abdominis muscle. An intact inferior epigastric vessel bundle served as an anatomical landmark and a point of orientation. The vas deferens/round ligament, which crossed the surgical field in almost rectangular fashion, was exposed (Fig. 2c, d). The external iliac vessels were fully exposed by careful electrocautery and removal of perivascular adipose tissue and lymphatic tissue (Fig. 2d).

### Working space areas

**Fig. 3:**
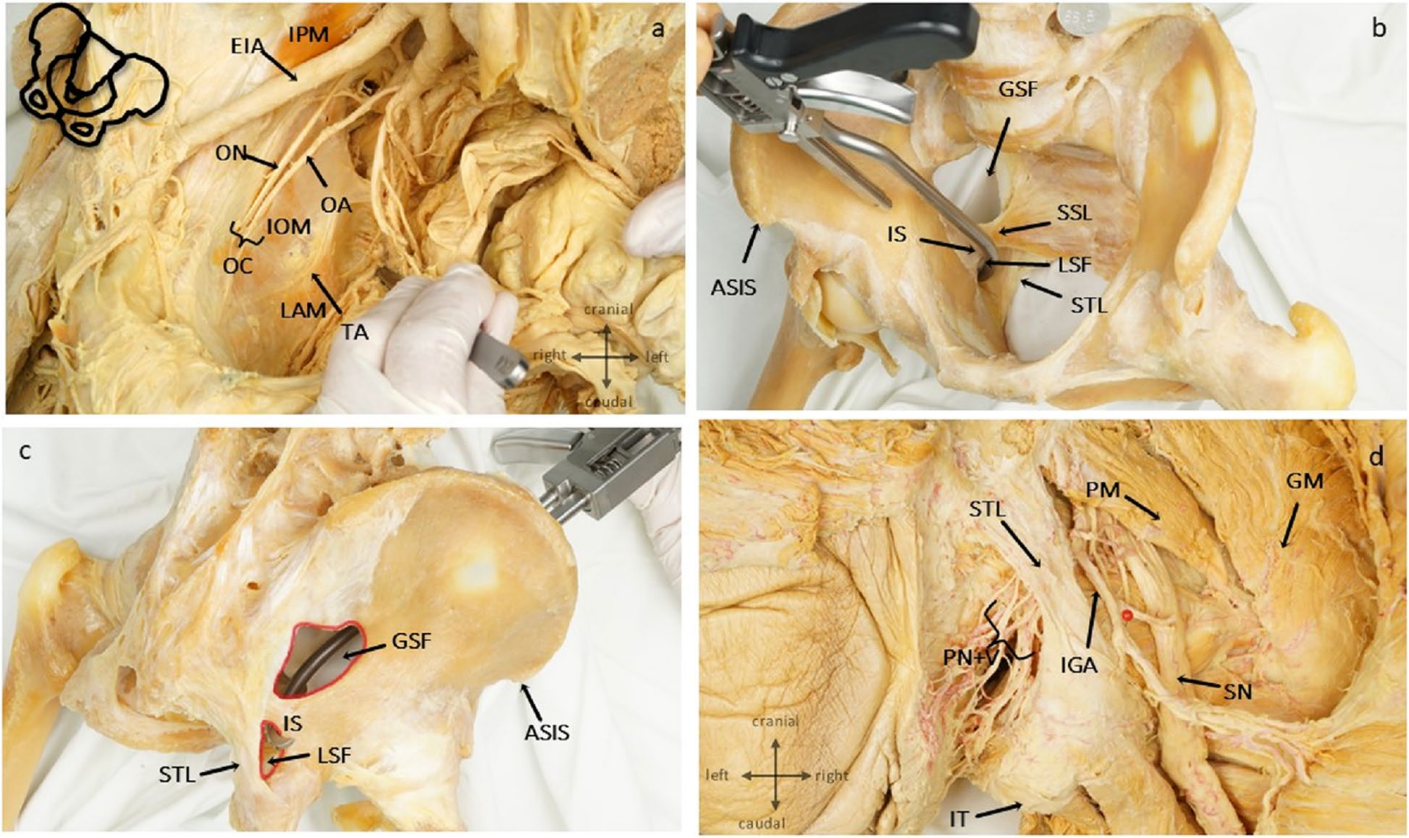
Anatomic dissection of relevant structures: **a** Topography of the pelvic visceral vessels and nerves. The quadrilateral plate is covered by the internal obturator muscle and the tendinous arch of levator ani (TA). **b** A collinear reduction clamp is placed into the lesser sciatic foramen (LSF) and on top of the acetabular roof to compress an anterior column fracture. The hook is placed close to the ischial spine (IS), a homan retractor can also be positioned here to retract bladder and peritoneum (GSF greater sciatic foramen, SSL sacrospinous ligament, STL sacrotuberal ligament. **c** The same anatomic specimen: from posteriorly the relation of ischial spine, greater and lesser sciatic notch is displayed. **d** A soft tissue specimen demonstrates the proximimty of the instrument (hook or homan retractor) to the pudendal canal and the clunium nerve bundle (PC). The inferior gluteal artery (IGA) and the sciatic nerve (SN) take their route distally in line with the sacrotuberal ligament until the vessel crosses the nerve at the level of the ischial tuberosity (IT). (PM piriformis muscle, GM gluteus medius muscle)

The selected access route enabled the surgeon to develop three working spaces or “surgical windows”:

- 1. Lateral window: The skin incision was gently expanded laterally and cranially to further mobilize the peritoneum and expose the psoas major fascia. In the event of an ilium fracture, the fascia was incised and the iliopsoas muscle was undermined subperiosteally. The genitofemoral nerve was identified along the psoas major muscle, lateral to the external iliac vessels.
- 2. Medial window: The medial window extended between the iliopsoas muscle and the external iliac vessels, which were protected and mobilized by a rubber band/vessel loops (Fig. 2e). Fractures extending through the iliopectineal line and the roof of the acetabulum could be addressed, and screw fixation into the posterior column performed.
- 3. Median window: This window was located medial to the external iliac vessels and could be established as in the modified Stoppa approach. The vas deferens/round ligament was retracted laterally, while the urinary bladder was carefully mobilized medially to gain access to the inner wall of the acetabulum and the quadrilateral plate (Fig. 2e). A Hohmann retractor was placed anterior to the superior pubic ramus to retract the muscles of the abdominal wall. The iliopectineal ligament was incised from the pubic symphysis into the dorsal aspect. At a distance of 6–8 cm from the pubic symphysis, a so-called corona mortis regularly crossed the line of incision (Fig. 2f). This vessel was present in some cases and usually formed a venous anastomosis between the external iliac vein and the obturator vein. In rare cases, we found an arterial anastomotic connection between the external iliac artery and the obturator artery. In the event of severe dislocation of the quadrilateral plate, this vessel may be disrupted by the injury. Therefore, it is important to identify and ligate these vascular structures. Ligation does not compromise blood supply of the obturator region and inner thigh because of the abundant pelvic vascular collaterals in this region. The exposure was concluded by elevating the iliopsoas and internal obturator muscles with a rasp. The obturator nerve courses in the cranial aspect of its accompanying vessels and was easily identified as it entered the obturator canal just below the superior pubic ramus (Fig. 2f). An anatomical dissection gives an overview of critical structure anatomy (Fig. 3a).

### Clinical application of the anterior transmuscular approach

#### Fracture reduction

A Hohmann retractor was inserted with the tip anchored posterior to the ischial spine in the lesser sciatic notch, to retract the bladder, other viscera, and the abdominal wall medially. The retractor was advanced carefully, the levator ani muscle was divided bluntly, and the tip of the retractor was placed beneath the ischial spine (Fig. 3b-d). Bleeding from the corona mortis can be safely prevented by dissection and ligation of the vessel(s). Visualization and management of the obturator vessels can be more challenging because they extend much deeper into the operating field and are accompanied by the obturator nerve which must be preserved.

Fracture reduction was mainly achieved by traction on the injured hip and compression in two main directions: To reduce anteroposterior displacement, a hook or a collinear reduction clamp was placed next to the lesser sciatic notch (Fig. 3b, c). Dissection and blunt piercing of the levator ani muscle had to be carried out carefully because the ischioanal fossa, containing the pudendal nerve and vessels, extends dorsocaudally (Fig. 3d) (for a detailed anatomical description see [10]). Mediolateral displacement was addressed by pushing outward and against the quadrilateral plate with a ball spike. In most cases, a pre-contoured plate supporting the quadrilateral surface was inserted first. Final fracture reduction was achieved by placing the ball spike and reduction clamps anteroposteriorly and mediolaterally over the plate. Wound closure included meticulous reconstruction of the abdominal wall.

#### Postoperative care and assessment

Full weight-bearing was advised as early as possible. Patients were discharged after wound healing and sufficient mobilization. The medical records of all patients operated on by the new approach between February 2019 and July 2021 were inspected in order to evaluate demographics, perioperative and intraoperative parameters.

All patients received a postoperative questionnaire, including the Harris hip score, the EQ-5D-5, the Merle d’Aubigné score, and the Postel score. After consulting a senior radiologist, all fractures were classified according to Letournel’s classification system. Fracture reduction was assessed on postoperative CT scans. Matta scores and maximum initial fracture displacement were registered.

#### Patient demographics

Twenty consecutive patients (mean age 68.8 ± 19.1 years), seven (35%) female and 13 (65%) male, were included in the study. The mean BMI was 25.5 (± 4.4) kg/m2. The fracture patterns were as follows: six (30%) anterior column and posterior hemitransverse fractures, six (30%) T-type fractures, five (25%) both-column fractures, one (5%) transverse, one (5%) transverse posterior wall, and one (5%) anterior column fracture.

Five (25%) patients had isolated acetabular fractures while 15 (75%) had experienced multiple injuries- The most common accompanying injury was a pelvic ring fracture, which was found in nine (45%) cases. Other accompanying injuries included those of the extremities in six (30%), pneumothorax in two (10%), lung contusion in one (5%), pleural effusion in one (5%), rib fracture in three (15%), spinal injury in two (10%), craniocerebral injury in two (10%), and abdominal injury in two (10%) patients. Table 1 provides a summary of epidemiologic data.

**Table 1:** Patient demographics categorial variables are given as absolute numbers (percentages). Noncategorial variables are given as mean ± SD

Patient demographics (*n* = 20)	
**Gender**	
Male	13 (65%)
Female	7 (35%)
**Age:**	68,8 ± 19,1 (21—94)
**Isolated acetabular fracture:**	5 (25%)
**Multiple injuries:**	15(75%)
Pelvic ring fracture	9 (45%)
Injury of extremity	6 (30%)
Pneumothorax	2 (10%)
Lung contusion	1 (5%)
Pleural effusion	1 (5%)
Rib series fracture	3 (15%)
Spinal injury	2 (10%)
Craniocerebral injury	2 (10%)
Injury of the abdomen	2 (10%)
**Mechanism of Injury:**	
Fall at same level	11 (55%)
Fall ≤ 3 m	2 (10%)
Fall ≥ 3 m	2 (10%)
Car accident	1 (5%)
Bicycle accident	2 (10%)
Heavy weight fallen on patient	2 (10%)
**Fracture type (Letournel classification):**	
Anterior column	1 (5%)
Transverse	1 (5%)
Anterior column + posterior hemitransverse	6 (30%)
Transverse posterior wall	1 (5%)
T-type	6 (30%)
Both columns	5 (25%)
**Periprosthetic fracture:**	5 (25%)
**Initial fracture displacement:**	
5—10 mm	11 (61%)
11—20 mm	6 (33%)
21—30 mm	1 (6%)
≥ 31	0 (0%)

## Results

The mean interval between injury and surgery was 10.5 ± 6.3 days, the mean operating time 209.9 ± 54.9 min, the mean incision length 11.4 ± 2.2 cm, and mean blood loss 1022.5 ± 1005.3 ml. Intraoperative complications occurred in three (15%) patients. These included vascular damage in two (10%) cases (injury to the corona mortis and the external iliac vessels). Both lesions could be managed successfully intraoperatively. Perioperative parameters are shown in Table 2.

**Table 2:** Perioperative data

Patient No	Age (years)	ASA-Level	Additional KL	Time to operation (days)	Total stay in hospital (days)	Operative time (minutes)	Incision length (cm)	Blood loss (ml)	Intraoperative Complications	Postoperative Complications
1	55	2		5	23	256	10	2400	Corona mortis injury	
2	21	1	Yes	3	9	267	15	1600		
3	47	2	Yes	5	68	208	10	500		SNI, UTI
4	83	3		19	42	244	16	1000	NSTEMI	DVT, PE, DI, H, GIB, WHD
5	38	3		10	16	129	10	500		
6	63	2		2	8	171	12	800	Ext. iliac vessels injury	WHD
7	68	2		12	38	181	15	400		WHD, P, UTI, SE
8	68	3	Yes	25	38	252	16	1150		WHD, P, UTI, SE
9	87	3		4	11	276	10	1800		
10	63	3		19	25	140	10	500		
11	92	3		9	22	197	10	800		H
12	82	2		15	45	175	10	400		WHD, UTI, B
13	59	2		9	41	302	11	4600		SE, EE
14	82	2		13	21	99	10	300		D
15	82	3		6	19	206	10	1000		
16	86	2		15	13	163	10	500		D
17	82	3		4	22	216	12	700		UTI
18	57	2		13	24	210	10	300		FNI
19	67	3	Yes	16	31	216	10	400		FNI
20	94	3		6	18	289	10	800		WHD, NSTEMI, P
**Mean**	68,8			10,5	26,7	209,9	11,4	1022,5		
**SD**	19,1			6,3	14,9	54,9	2,2	1005,3		

*KL* Kocher—Langenbeck approach, *SNI* Sciatic nerve injury, *UTI* Urinary tract infection, *DVT* Deep vein thrombosis, *PE* Pumonary embolism, *DI* Deep infection, *GIB* Gastrointestinal bleeding, *WHD* Wound healing disorder, *P* Pneumonia, *B* Bleeding, *SE* Scrotal edema, *EE* Extremity edema, *D* Delirium, *FNL* Femoral nerve injury

Of 20 patients, four (20%) required further surgery via a posterior Kocher-Langenbeck approach due to accompanying posterior pathologies. One (5%) patient received a hip arthroplasty shortly after the anterior acetabular surgery.

Postoperative surgery-related adverse events included hemorrhage from the inferior epigastric artery, which required emergency coil embolization and additional exploratory laparotomy due to persistent bleeding in one (5%) patient. The patient had a history of various urogenital surgeries due to bladder cancer, resulting in considerable scar tissue which complicated the operation in the acetabulum. Postoperative neurological symptoms were noted in three (15%) patients; two patients experienced injury to the femoral nerve and one had an injury of the common fibular nerve.

A significant hematoma was noted in two (10%) patients. One of these involved retention of serous fluid and required CT-guided puncture; the condition resolved fully. A further six (30%) patients had slight wound healing disorders which resolved completely before discharge. One of these (5%) developed an early deep infection of the implanted material, which could be managed with antibiotic treatment.

One (5%) patient had a deep vein thrombosis and pulmonary embolism with pleural effusion, and was successfully treated with percutaneous transluminal angioplasty (PTA) stents as well as full anticoagulation with heparin.

Other general and in-hospital complications included anemia in five (25%) patients, urinary tract infection in five (25%), pneumonia in three (15%), scrotal or extremity edema in three (15%), NSTEMI in one (5%), delirium in two (10%), and gastrointestinal bleeding in one (5%) patient. No inguinal or abdominal wall hernia, and no obturator nerve injury or atrophy of the rectus abdominis muscle was observed.

Clinical results are summarized in Table 3. Radiological analysis of preoperative X-rays and CT scans showed that initial fracture displacement was 5–10 mm in 11 (61%) patients, 11–20 mm in six (33%), and 21–30 mm in one (6%) patient (mean displacement 10.1 ± 7.6 mm). Preoperative CT scans of the hip were not available in two cases. According to the Matta scoring system, the quality of fracture reduction on CT scans was “anatomical” (≤ 1 mm) in 12 (60%), “imperfect” (2-3 mm) in four (20%), and “poor” (> 3 mm) in four (20%) patients [11].

**Table 3:** Radiological and clinical results. The radiological outcome and clinical results are presented in numbers (percentage)

Radiological and clinical results	
**Quality of Reduction (Matta) (*n* = 20)**	
Anatomical (< 1 mm)	12 (60%)
Imperfect (2–3 mm)	4 (20%)
Poor (> 3 mm)	4 (20%)
**Merle d’Aubigné Postel Score (*n* = 9)**	
Excellent (17–18)	0 (0%)
Good (15–16)	3 (33%)
Fair (13–14)	5 (56%)
Poor (< 13)	1 (11%)
**Harris Hip Score (*n* = 9)**	
Excellent (90–100)	3 (33%)
Good (80–89)	0 (0%)
Fair (70–79)	1 (11%)
Poor (< 70)	5 (56%)
**EQ-5D-5L Index (*n* = 9)**	0.57 ± 0.32

Not all members of the elderly patient cohort were available for follow-up. Only nine (45%) patients could be followed for a mean period of 8.5 months. Three (15%) patients with various medical comorbidities died postoperatively due to unrelated causes over an average period of three months. One (5%) patient developed a femoral head necrosis leading to total hip replacement four months after discharge. This may be interpreted as a typical complication of the posterior Kocher-Langenbeck approach that the patient had received in addition to anterior stabilization approximately four months earlier. Another patient underwent total hip replacement due to osteoarthritis and related symptoms within eight months after the surgery.

In the nine patients who were available for follow-up, Merle d’Aubigné-Postel scores revealed a good outcome in three cases, a poor outcome in five, and an unsatisfactory outcome in one case. The Harris hip score was excellent in three cases, fair in one, and poor in five cases. The mean EQ-5D-5L health index score was 0.57 ± 0.32. All wounds had healed uneventfully.

### Comparison of ATI, pararectus approach, stoppa approach (Table 4)

**Table 4:** Summary of characteristics and advantages/disadvantages of three anterior approaches

	**Skin incision**	**Route through abdominal wall**	**Vessels crossing approach superficially**	**Vessels and nerves exposed close to bone**	**Topography of approach/structures at risk**	**Exposure of bony pathology**	Advantages/Disadvantages
**ATI**	Curved or straight, 8–15 cm	Directly through 3 abdom. muscles	Inferior epigastric bundle	External (and internal) iliac vessels, Round Ligament, vas deferens, femoral nerve, ilioinguinal nerve, obturator nerve and vessel, corona mortis	Directly over acetabular roof- peritoneum has to be mobilized medially Less tension of abdominal wall muscles, all critical structures visualized	Entire Iliac wing, full exposure of anterior column, tectum, quadrilateral plate, superior pubic ramus	Most direct approach, least tension on abdominal wall muscles and soft tissue, closure in 3 layers ensures sufficient healing / great vessels may be injured
**Pararectus Approach**	Curved or straight, 8–15 cm	Through pararectal fascia	Inferior epigastric bundle	External (and internal) iliac vessels, Round Ligament, vas deferens, femoral nerve, ilioinguinal nerve, obturator nerve and vessel, corona mortis	Medial to acetabular roof- peritoneum has to be mobilized, tension of abdominal muscles may hinder full exposure of iliac wing, all critical structures visualized	Full exposure of entire iliac wing and complete anterior column only possible with longer skin incision, good access to tectum, quadrilateral plate, superior pubic ramus	Direct approach with good visualization of all critical structures, abdominal wall muscles need to be mobilized laterally. Closure in one layer of pararectal fascia is critical to avoid complications i.e. pararectal or juxasymphyseal hernia
**Modif. Stoppa Approach**	Pfannenstiel	Midline, through rectus sheeth, occasionally detaching ipsilateral rectus belly Peritoneum usually hardly visualized/left cephalad	None superficially	Corona mortis, obturator nerve and vessel	Medial to acetabular roof- tension of abdominal muscles hinder full exposure of anterior column, tension on iliac vessels though not visualized but elevated/retracted	Limited exposure of acetabular roof/ anterior column, full exposure of superior pubic ramus, additional incision necessary for iliac wing fractures	Surgically easiest route, no exposure of great vessels, vision limited due to retracted abdominal wall muscles, closure critical in single layer to avoid pararectal, juxtasymphyseal hernia

## Discussion

Traditionally, acetabular surgery is associated with poor clinical outcomes and complications, imposing high demands on the surgeon in terms of skill and experience. Although there is no consensus about a definite approach for specific fracture types, the large majority of orthopedic surgeons use the anterior approach when major displacement is accompanied by an anterior pathology. The ilioinguinal approach has been used for a long time and has served almost as the sole viable option. The only alternative is the extended iliofemoral approach. However, the major disadvantage of the latter is insufficient exposure of the quadrilateral plate and the complex surgical technique needed for most fractures [12].

Therefore, less invasive anterior approaches have become increasingly popular. The modified Stoppa approach, first described by Hirvensalo, proved to be a feasible alternative. It is less time consuming, causes fewer complications, and provides better exposure for reduction [5, 6, 12–14]. In many cases, the anterior approach alone is not sufficient to address a pathology of the iliac wing or fix the posterior column indirectly; a second incision of the iliac wing is needed. Ruchholtz et al. described a comprehensive minimally invasive procedure, and named it TIMI or the two-incision minimally invasive approach; the latter provided favorable results and was associated with few complications in an elderly population [15].

Given that these incisions rather flank the anatomical region of interest, Keel adopted a common approach in general surgery and urology, namely the pararectus approach directly over the acetabulum [7]. Entering the intrapelvic cavity through an incision of the fascial strip between the rectus sheath and the insertion of the lateral abdominal wall muscles allowed direct exposure of the iliopsoas fascia, the neurovascular structures and the bony anatomy of the ilium, the acetabular roof, the pubic ramus and the quadrilateral plate through a single approach. This procedure rapidly became a safe and effective alternative to the traditional ilioinguinal approach as well as the modified Stoppa approach [16–18]. The method is associated with the potential complication of a pararectal hernia, which is most likely due to the fact that closure of the incision is highly dependent on accurate adaptation of the fascial strips of the transversus abdominis muscle and the rectus sheath.

Recently, Chen et al. proposed the use of a transmuscular approach involving transection of the abdominal wall musculature lateral to the rectus sheath, and termed it the “suprailioinguinal approach” [8]. We adopted this access route, hypothesizing that multilayered reconstruction of the abdominal wall muscles and fascial structures provides better healing of the access, while the incision is centered over the surgical target.

Anatomical dissections focused on the abdominal wall anatomy revealed that an incision lateral to the rectus sheath may allow for precise dissection of all three layers of the abdominal wall muscles, including their fascial coverings. Incision of each fascia in line with the course of the muscle fibers serves as a muscle-sparing technique, while the continuity of the wall is nearly preserved. A clinical review of our patients in regard of wound healing and incisional hernia confirmed the proof of this concept. With the exception of one case of substantial bleeding from the epigastric vessels, probably due to insufficient ligature, we registered no adverse events related to the access route.

One of the key parameters especially in elderly patients is wound healing. Here we found minor problems with wound healing in 30%, which seems relevant. Nevertheless, only in one case a revision was necessary and all other cases healed within appropriate time frames. With respect to the fact that wound healing is generally a c ritical issue in the elderly population we did not feel there was an increased rate of wound healing associated problems with the new approach.

In addition to a thorough anatomical description of the surgical approach, anatomical dissection allowed for complete exposure of those structures not usually dissected in the operating room. A key step during exposure and fracture reduction is the placement of a retractor and a reduction forceps deep into the pelvis, namely in the posterocaudal aspect of the ischial spine. This area can only be exposed in part, and never from the posterior aspect. At this site we were able to penetrate the pelvic floor/levator ani muscle gently by blunt dissection along the quadrilateral plate. Clinically, this maneuver led to no significant complications. However, care should be taken to avoid collateral pelvic floor damage. The same applies to the awareness of neurovascular structures in close proximity to the lesser ischial foramen. Thus, placement of a hook into the lesser sciatic notch should be performed carefully and close to the bone in order to prevent injury to the pudendal neurovascular structures that run around the ischial spine and extend throughout the ischioanal fossa within the pudendal canal (Alcock).

Initially, the new approach was performed by a multidisciplinary surgical team consisting of an orthopedic surgeon, a gynecological oncologist, and a urologist. Its application was subject to a learning curve. The absence of major complications was probably because the interdisciplinary strategy took the unique anatomical features and major pitfalls of the access route into account.

A detailed evaluation of the approach revealed that it provided satisfactory clinical and radiological results. The approach was used for all both-column, anterior column and posterior hemitransverse, and T-type fractures with the main displacement in the anterior column or the quadrilateral plate. The achieved reduction was similar to the data reported recently by other authors [13, 18]. Intraoperative features such as blood loss and operating times were comparable to those reported for other modern anterior approaches. Perioperative complications were equivalent to those associated with other approaches, and the postoperative investigation revealed a functional outcome in line with those described in other studies [12, 17].

We noted a relatively high number of femoral nerve injuries, which all resolved slowly. We attribute these to the fact that the contents of the lacuna musculorum is mobilized to an extent that palsy of the femoral nerve can occur by tractrion with homann retractors.

Especially when using an additional posterior approach, chondral damage led to early degenerative arthritis and ultimately required early conversion to hip replacement [2]. The posterior approach and surgical hip dislocation make vascular damage of the head- supplying structures more likely- therefore it seemed not surprising that the rate of hip replacments after an. Additional posterior approach was high. The advanced age of the patient cohort did not permit a comprehensive postoperative follow-up. Although the overall results were encouraging, we lack long-term evidence of successful treatment. Continued application of the new method is expected to provide robust long-term data and confirm its benefits.

It must be considered as a feature of following up elderly patient cohorts that not all of the initially included patients could be seen for a regular post-operative check-up.

Resuming surgical considerations as well as clinical outcomes in elderly patients we have come to the conclusion that general patient conditions and fitness (i.e. comorbidities) play the most important role with regards to their outcome. Most patients we treat with acetabular fractures are geriatric patients today, therefore the invasiveness of approaches needs to be well considered. Clinically most patients we treated displayed disappointing results when compared to radiological measured like fracture reduction. All such evaluations must be interpreted with care- one must consider the full spectrum of complications with any approach to restoring hip mobility. Hip replacemement surgery has a high complication rate if standard fixtion methods of the acetabular component is impossible because of acetabular fracture [19]. In addition conversion rates of up to 12% are reported following surgical reconstruction in acetabular fractures of the elderly [20]. We therefore propose to reconstruct the acetabular cup in most cases.

## Conclusions

In summary, the new anterior transmuscular intrapelvic (ATI) approach was a versatile and effective method to address anterior pathologies in acetabular fractures. Relevant anatomical structures and landmarks related to the ATI procedure are highlighted by the approach and facilitated the modified access route. Despite the learning curve, we believe that experienced acetabular surgeons will be able to enhance their skills in terms of anatomic reduction and shorter operating times by the use of the ATI method.

## Data Availability

Data generated or analysed during this study are included in this published article in all publications are solely those of the individual author(s) and contributor(s) and not of MDPI and/or the editor(s). MDPI and/or the editor(s) disclaim responsibility for any injury to people or property resulting from any ideas, methods, instructions or products referred to in the content.
